# Ubiquitin-Specific Protease 11 Promotes Uric Acid-Induced Renal Tubular Epithelial Cell Apoptosis via Deubiquitinating P53 and Enhancing Mitochondrial Outer Membrane Permeabilization

**DOI:** 10.3390/biomedicines14081852

**Published:** 2026-08-18

**Authors:** Yingfeng Shi, Jinqing Li, Na Liu

**Affiliations:** Department of Nephrology, Shanghai East Hospital, School of Medicine, Tongji University, Shanghai 200120, China; shiyf_tongji@163.com (Y.S.); xxxlizi@126.com (J.L.)

**Keywords:** chronic kidney disease, ubiquitin-specific protease 11, apoptosis, p53, mitochondria

## Abstract

**Background/Objectives:** Chronic kidney disease (CKD) has emerged as a critical global health challenge, driven by its high prevalence and poor prognosis. Ubiquitin-specific protease 11 (USP11), a deubiquitinating enzyme, participates in DNA damage repair, cell-cycle regulation, and immune modulation, and has been shown to promote epithelial–mesenchymal transition in renal tubular epithelial cells (TECs) during CKD progression. However, whether USP11 also plays a critical role in the apoptosis of TECs remains to be elucidated. **Methods:** In this study, we demonstrate that USP11 deubiquitinates and stabilizes p53, thereby activating the p53-mediated canonical mitochondrial pathway of apoptosis in cultured human proximal TECs (HK-2). **Results:** In uric acid (UA)-stimulated HK-2 cells, activated p53 subsequently increases mitochondrial outer membrane permeabilization (MOMP), leading to the release of cytochrome c from the mitochondria into the cytosol, which, in turn, activates caspase 9 and caspase 3, and ultimately promotes TEC apoptosis. Inhibition of USP11 with siRNA transfection or mitoxantrone (MTX), a small-molecule inhibitor of USP11, downregulates p53 expression and blocks cytochrome c release into the cytosol, thereby significantly reducing TEC apoptosis. In vivo, both conditional genetic knockout and pharmacological inhibition of USP11 effectively reduce apoptosis of tubular cells and ameliorate kidney injury in a mouse CKD model of hyperuricemic nephropathy (HN). **Conclusions:** In summary, our results underscore the pivotal role of USP11 in TEC apoptosis during CKD progression and suggest that targeting USP11 represents a potential therapeutic approach for CKD patients.

## 1. Introduction

Chronic kidney disease (CKD) refers to a long-term condition characterized by persistent abnormalities in kidney structure or function. It often develops without obvious early symptoms, progresses gradually over time, and is generally irreversible [1,2]. Owing to its increasing prevalence and substantial impact on morbidity and mortality, CKD has emerged as a major global public health challenge. Globally, it is estimated that approximately 850 million people are affected by CKD, with around 4 million individuals requiring kidney replacement therapy due to kidney failure [1]. It is projected that by 2050, CKD will become the fifth leading underlying cause of death worldwide [1]. CKD arises from multiple etiologies, with common contributors including diabetes mellitus, hypertension, hyperuricemia, and various glomerular disorders [1,3]. As the disease progresses, CKD patients may eventually progress to end-stage renal disease (ESRD) and are also associated with a significantly increased risk of cardiovascular events and mortality [3,4]. Therefore, gaining insight into the molecular and cellular mechanisms of CKD initiation and development is crucial for slowing disease advancement and improving clinical outcomes.

In the progression of CKD, the apoptosis of tubular epithelial cells (TECs) is regarded as a pivotal event that drives both nephron loss and interstitial fibrosis [5,6]. Apoptotic TECs detach and obstruct the tubular lumen and breach the basement membrane barrier, thereby causing permanent atrophy of functional nephrons. More importantly, rather than dying “silently”, apoptotic TECs actively release numerous signaling molecules that drive the direct activation of interstitial fibroblasts. Studies have reported that injured TECs release abundant exosomes, which, upon uptake by renal interstitial fibroblasts, markedly promote their proliferation [7]. Recent evidence also indicates that in CKD, apoptotic bodies derived from apoptotic TECs accumulate in the renal interstitium due to impaired clearance. These apoptotic bodies carry bioactive molecules, such as damage-associated molecular patterns (DAMPs), cytokines, growth factors, and profibrotic microRNAs, that can directly drive fibroblast activation and extracellular matrix (ECM) deposition [8]. Furthermore, the massive release of these bioactive molecules remodels the renal interstitial microenvironment, triggering macrophage infiltration and sterile inflammation, thereby establishing a vicious “apoptosis-inflammation-fibrosis” cycle [8,9]. Therefore, apoptosis of TECs is critically involved in both the initiation and progression of CKD.

p53-mediated apoptosis is a form of intrinsic cell death involving the mitochondrial pathway and represents one of the most important and classical regulatory mechanisms of programmed cell death [10,11]. Upon exposure to cellular stress or pathological stimuli, such as DNA damage, radiation, or oncogenic signals, p53 becomes activated and functions as a transcription factor to upregulate the pro-apoptotic protein Bax while downregulating the anti-apoptotic protein Bcl-2 [10]. This shift disrupts the balance between cell survival and death, leading to increased mitochondrial outer membrane permeabilization (MOMP) [12]. Subsequently, due to enhanced membrane permeability, cytochrome c is released from the mitochondria into the cytoplasm, where it associates with Apaf-1 and ATP to form the apoptosome [13,14]. This complex then recruits and activates caspase-9, which, in turn, initiates a cascade of executioner caspases, particularly caspase-3, ultimately resulting in DNA fragmentation and cellular disassembly, thereby completing the apoptotic process [13,14]. In this regulatory pathway, p53 acts as the “decision-maker”, the mitochondria serve as the “switch”, and caspases function as the ultimate “executioners”, each playing its distinct role. As the initiating regulator in this apoptotic cascade, the activation of p53 is particularly critical.

Ubiquitin-specific protease 11 (USP11) is a cysteine protease belonging to the deubiquitinase (DUB) family. Its primary function is to remove ubiquitin molecules from substrate proteins, thereby enhancing their function or prolonging their half-life, ultimately regulating protein stability, localization, and activity. USP11 is widely involved in various cellular processes, including DNA damage repair, cell-cycle regulation, and apoptosis [15]. USP11 has been demonstrated to play important roles in various fibrotic diseases and is also capable of promoting the progression of kidney fibrosis [16,17]. In our previous study, we demonstrated that USP11 deubiquitinates and activates epidermal growth factor receptor (EGFR), thereby promoting partial epithelial-to-mesenchymal transition (EMT) of renal tubular epithelial cells during kidney fibrosis [17]. In addition, TGF-β type II receptor (TGFβRII) has been identified as another ubiquitination substrate of USP11, through which USP11 promotes renal tubular cell senescence and fibrosis [16]. The aforementioned studies have primarily focused on partial EMT and cellular senescence in TECs. Therefore, whether USP11 also plays a significant role in the apoptosis of TECs remains unknown. If so, whether USP11 is associated with p53, the decision-maker of apoptosis, requires further investigation and confirmation.

In the current study, we found that USP11 promotes apoptosis of TECs, thereby contributing to kidney injury and the progression of CKD. In this process, USP11 deubiquitinates p53 and stabilizes its protein structure, which, in turn, activates p53 and its canonical mitochondrial pathway of apoptosis. Pharmacological intervention with USP11-specific inhibitor mitoxantrone (MTX, an FDA-approved anticancer drug) or genetic knockout of USP11 significantly reduces TEC apoptosis. Therefore, from a novel perspective, this study further demonstrates the importance of USP11 in the progression of CKD.

## 2. Materials and Methods

### 2.1. Antibodies and Reagents

Antibodies to Cleaved caspase 3 (#9664) and ubiquitin (#3933) were purchased from Cell Signaling Technology (Dancers, MA, USA). Antibodies to GAPDH (sc-32233) and USP11 (sc-365528) were purchased from Santa Cruz Biotechnology (Santa Cruz, CA, USA). Antibody to USP11 (ab109232) was purchased from Abcam (Cambridge, MA, USA). Antibody to Bax (GB114122) was purchased from Servicebio (Wuhan, China). Antibody to Bcl-2 (BS1511) was purchased from Bioworld (Los Angeles, CA, USA). Antibodies to Cleaved caspase 9 (10380-1-AP), Tom20 (11802-1-AP), NGAL (31721-1-AP), Kim-1 (30948-1-AP) and Cytochrome c (10993-1-AP) were purchased from Proteintech (Wuhan, China). Antibody to p53 (A3185) was purchased from Abclonal (Wuhan, China). MTX, Cycloheximide (CHX) and MG132 were purchased from Selleckchem (Houston, TX, USA). FITC and Texas Red for immunofluorescent staining were purchased from Thermo Fisher Scientific (Waltham, MA, USA). USP11 siRNA and p53 siRNA were purchased from GenePharma (Shanghai, China). Potassium oxonate and adenine were purchased from Macklin (Shanghai, China). Lipofectamine 3000 was purchased from Invitrogen (Grand Island, NY, USA). Uric acid (UA), secondary antibodies for Western blot, and all other chemicals were purchased from Sigma (St. Louis, MO, USA).

### 2.2. Cell Culture and Treatment

Human proximal tubular epithelial cells (human kidney-2 cells, HK-2) were purchased from ATCC (Manassas, VA, USA) and cultured in a 1:1 mixture of Dulbecco’s modified Eagle’s medium (DMEM) and F12 containing 10% fetal bovine serum (FBS), 1% penicillin and streptomycin in an atmosphere of 5% CO_2_ and 95% air at 37 °C. After passing the primary HK-2 cells for three generations, we obtained a stable phenotype to start the formal experiments. We stimulated with UA and found that UA upregulated USP11 expression in HK-2 in both dose-dependent and time-dependent manners. Among the conditions tested, stimulation with 800 μM UA for 36 h yielded the most pronounced effect and was therefore selected as the optimal condition for subsequent cellular assays. UA-stimulated HK-2 cells were starved for 24 h with DMEM/F12 containing 0.5% FBS and then were pretreated with MTX (0, 1, 5, 10 μM) for 1 h. After that, cells were exposed to UA (800 μM) for an additional 36 h in the presence of different doses of MTX (0, 1, 5, 10 μM) before cell harvesting. To verify the proteasome degradation of p53, starved HK-2 cells were exposed to MG132 (5 μM) in the presence or absence of UA (800 μM) or USP11 siRNA for an additional 36 h. CHX (10 μg/mL) was applied for 0, 3, 6, 9, or 12 h in HK-2 cells under UA stimulation, with or without USP11 siRNA transfection. All in vitro experiments were repeated at least four times.

### 2.3. Animals and Treatment

All animal work was performed at Tongji University School of Medicine (Shanghai, China). Male C57/black mice (Shanghai Super-B&K laboratory animal Corp. Ltd., Shanghai, China) that weighed 20–25 g were housed under a 12 h light-dark cycle with food and water supplied ad libitum. All animals were acclimated to this environment for 7 days before experiments. The CKD model of hyperuricemic nephropathy (HN) was established by oral administration of a mixture of adenine (0.14 g/kg) and potassium oxonate (2.1 g/kg) for mice daily consistently for three weeks. To investigate the effect of MTX on CKD progression, mice in HN model were intraperitoneally injected with two different doses of MTX (0.04375 mg/kg and 0.0875 mg/kg) in saline every day. The sham group was injected with an equal volume of saline as a control. After three weeks of HN, the animals were sacrificed by exsanguination under anesthesia with inhaled 5% isoflurane in room air and the kidney samples were collected for protein analysis and histological examination. The animal protocol was reviewed and approved by the Institutional Animal Care and Use Committee at Tongji University (Shanghai, China).

### 2.4. USP11 Conditional Knockout Mouse Model

To generate compound mice USP11*^fl/fl^*Cdh16-Cre^+/−^(USP11-cKO), heterozygous USP11^+/*flox*^ mice, purchased from shanghai model organisms (Shanghai, China), were crossed with cadherin 16-cre mice (Tg(Cdh16-cre)91Igr/J, Stock No.: 012237), purchased from the Jackson Laboratory (Bar Harbor, ME, USA). Both USP11-cKO mice and USP11-WT mice received a daily oral administration of a mixture of adenine (0.14 g/kg) and potassium oxonate (2.1 g/kg) for three weeks to establish HN model.

### 2.5. Transfection of USP11 and p53 siRNA

Two independent siRNAs targeting USP11 (USP11 siRNA #1 and USP11 siRNA #2) and one siRNA targeting p53 were synthesized by GenePharma (Shanghai, China). Both USP11 siRNA #1 and USP11 siRNA #2 were transfected into HK-2 cells, and USP11 siRNA #2 was selected for subsequent experiments based on its higher knockdown efficiency as determined by Western blotting (Supplementary Figure S2). Transfection of siRNA was performed according to the manufacturer’s protocol, respectively. Briefly, HK-2 cells were seeded to 70–80% confluence in the antibiotic-free medium and grown followed by transfection with USP11 or p53 siRNA (60 pmol) using Lipofectamine 3000. In parallel, scrambled siRNA (60 pmol) was used as a control for off-target changes in HK-2 cells. After transfection, the medium was changed to DMEM with F12 containing 0.5% FBS for starvation and then cells were incubated with or without UA (800 μM) for an additional 36 h before being harvested for analysis. All of the in vitro experiments were repeated for at least four times.

### 2.6. Transfection of USP11 and p53 Plasmids

The plasmid USP11-pcDNA 3.0 was synthesized by Genewiz (Shanghai, China) and p53-pcDNA 3.1 was synthesized by GenePharma (Shanghai, China). USP11 and p53 plasmids were transiently transfected into HK-2 cells seeded in 6-well plates using Lipofectamine LTX Reagent with PLUS Reagent (Invitrogen, Grand Island, NY, USA) according to the manufacturer’s instructions. Briefly, for each well, 2 μg of plasmid DNA was diluted in Opti-MEM reduced-serum medium and mixed with 2 μL of PLUS Reagent and 6 μL of Lipofectamine LTX Reagent. The transfection complexes were incubated at room temperature for 20 min and then added dropwise to the cells. After 6 h of transfection, the medium was replaced with DMEM/F12 supplemented with 10% FBS, and the cells were cultured for an additional 24 h prior to further analysis. All of the in vitro experiments were repeated for at least four times.

### 2.7. Co-Immunoprecipitation (Co-IP)

HK-2 cells grown in 10 cm dishes were washed with PBS, then lysed on ice for 30 min in 1 mL cold low-stringency lysis buffer. Lysates were centrifuged at 12,000× *g* for 10 min at 4 °C. The supernatant was collected and incubated with USP11 antibody, p53 antibody or IgG, followed by addition of 20 µL A/G PLUS-Agarose beads (SC-2003, Santa Cruz Biotechnology, Santa Cruz, CA, USA) with rotation at 4 °C overnight. Subsequently, the beads were washed five times with a 1 mL low-stringency lysis buffer between each wash at 2500 g for 5 min at 4 °C. The wash buffer was discarded, and 50 µL of 1× SDS-PAGE loading buffer was added to the immune complexes, which were then denatured at 100 °C for 10 min. Subsequently, the samples were processed for Western blot analysis.

### 2.8. Isolation of Mitochondria from HK-2 Cells

Mitochondria were isolated using a Cell Mitochondria Isolation Kit (C3601, Beyotime Biotechnology, Shanghai, China) according to the manufacturer’s instructions. Briefly, 1 × 10^7^ cells were harvested, washed with pre-cooled PBS, and resuspended in ice-cold mitochondrial isolation reagent supplemented with PMSF. The cell suspension was homogenized on ice using a Dounce homogenizer. The homogenate was then centrifuged at 600× *g* for 10 min at 4 °C to remove nuclei and unbroken cells. The resulting supernatant was carefully transferred to a new tube and centrifuged at 11,000× *g* for 10 min at 4 °C. After centrifugation, the supernatant was collected as the cytoplasmic fraction and stored at −80 °C for further analysis. The pellet (mitochondrial fraction) was washed and resuspended in storage buffer, and either used immediately for functional assays or stored at −80 °C.

### 2.9. Cell Lysis

Following the aforementioned cellular treatments, cells were washed with cold PBS. Cells were then collected and lysed in 120 μL of lysis buffer, which consisted of 20 mM Tris HCl (pH 7.4), 150 mM NaCl, 2 mM EGTA, 5 mM beta-glycerophosphate, 1 mM MgCl_2_, 1% Trton X-100, 1 mM sodium orthovanadate, 10 μg/mL protease inhibitors, 1 μg/mL aprotinin, 1 μg/mL leupeptin, and 1 μg/mL pepstatin. The cell lysates were then sonicated on ice for 12 s, followed by centrifugation at 4 °C at 12,000 r.p.m. for 15 min. Protein concentrations of the samples were then determined with use of a BCA Protein Assay Kit (Beyotime Biotechnology, Shanghai, China) using BSA as a standard. The samples were prepared for immunoblotting analysis.

### 2.10. Immunoblotting Analysis

Samples were all equilibrated to 20 μg and run on a 4–15% SDS-PAGE gel, transferred to a nitrocellulose membrane, and blocked in 5% nonfat biological grade powdered milk dissolved in 25 mM Tris HCl (pH 7.4), 137 mM NaCl, and 0.1% TWEEN20 (TBST) for 30 min. Blots were washed with TBST and incubated with primary antibody in 5% BSA with TBST for 1 h or overnight. The membranes were then washed three times at 10 min intervals with TBST prior to addition of secondary antibody for 1 h. Blots were developed with BeyoECL Plus Chemiluminescence Kit (Beyotime Biotechnology, Shanghai, China) according to the manufacturer’s instruction. The densitometry analysis of immunoblot results was conducted by using ImageJ software (version 1.52p, National Institutes of Health, Bethesda, MD, USA).

### 2.11. Immunofluorescent Staining

After different stimulation and treatment, HK-2 cells were fixed with 4% paraformaldehyde (PFA) for 15 min, typically at room temperature. Then, Triton X-100 (Dow Inc., Midland, MI, USA) was used to make the cell membrane permeable for 60 min, allowing antibodies to enter the cell interior. Cells were incubated with diluted primary antibody, overnight at 4 °C or according to the manufacturer’s recommendations. Cells were washed thrice in PBS and then incubated with secondary antibodies for 1 h at room temperature. Finally, the nuclei were stained with DAPI (1 μg/mL, Abcam) for 15 min at room temperature before visualization. Cells were observed with ZEISS Digital Camera for Fluorescence Microscopy (Carl Zeiss Microscopy LLC, White Plains, NY, USA).

### 2.12. Transmission Electron Microscopy (TEM)

For TEM observation of mitochondrial ultrastructure, cells were fixed with 2.5% glutaraldehyde in 0.1 M phosphate buffer (pH 7.4) at 4 °C for 2 h, followed by post-fixation with 1% osmium tetroxide at 4 °C for 1 h. The samples were then dehydrated through a graded ethanol series (30%, 50%, 70%, 80%, 90%, and 100%) and embedded in epoxy resin. Ultrathin sections were cut using an ultramicrotome, mounted onto copper grids, and double-stained with uranyl acetate and lead citrate. The sections were examined under a transmission electron microscope at an accelerating voltage of 120 kV, and mitochondrial morphology was observed and photographed.

### 2.13. Terminal Deoxynucleotidyl Transferase dUTP Nick End Labeling (TUNEL) Staining

Apoptosis was detected using a CF488 Tunel Cell Apoptosis Detection Kit (G1504-50T, Servicebio, Wuhan, China) according to the manufacturer’s instructions. Briefly, cells cultured on coverslips were washed with PBS, fixed with 4% paraformaldehyde for 20 min at room temperature, and permeabilized with 0.2% Triton X-100 for 5 min. After washing with PBS, 50 μL of TUNEL reaction mixture (prepared by mixing Recombinant TdT Enzyme, CF488-dUTP Labeling Mix, and Equilibration Buffer at a ratio of 2:5:50) was added to each sample and incubated at 37 °C for 60 min in a humidified chamber protected from light. The samples were then washed three times with PBS. Nuclei were counterstained with DAPI (1 μg/mL) for 5 min. The coverslips were mounted onto slides and observed under a fluorescence microscope. Apoptotic cells exhibited green fluorescence (CF488, excitation 490 nm, emission 515 nm).

### 2.14. Hematoxylin and Eosin (H&E) Staining and Tubular Injury Index

H&E staining was performed on paraffin-embedded kidney sections (4 μm). After deparaffinization and rehydration, sections were stained with hematoxylin for 5 min, rinsed, differentiated briefly in 0.3% hydrochloric acid in 70% ethanol, and blued in tap water. Sections were then stained with eosin for 1 min, dehydrated, cleared in xylene, and mounted with neutral resin. H&E-slide images were viewed with a microscope equipped with a digital camera. To assess the extent of tubular injury, morphologic damage (epithelial necrosis, luminal necrotic debris, and tubular dilation) was quantified using the following scale: none = 0, <30% = 1, 30–60% = 2 and >60% = 3.

### 2.15. Immunohistochemical Staining (IHC)

IHC was performed on paraffin-embedded kidney sections. Briefly, kidney tissues were fixed in 4% paraformaldehyde, dehydrated, and embedded in paraffin. Sections were cut at 4 μm thickness, deparaffinized in xylene, and rehydrated through graded ethanol. For antigen retrieval, sections were heated in citrate buffer (pH 6.0) or EDTA buffer (pH 8.0) using a microwave or pressure cooker for 10–15 min. Endogenous peroxidase activity was blocked with 3% hydrogen peroxide (H_2_O_2_) for 10 min at room temperature. Sections were then incubated with 5% bovine serum albumin (BSA) or normal goat serum for 30 min to block non-specific binding. The primary antibodies against NGAL and Bax were applied and incubated overnight at 4 °C. After washing with PBS, sections were incubated with HRP-conjugated secondary antibody for 30 min at room temperature. Signal was developed using diaminobenzidine (DAB) substrate, and sections were counterstained with hematoxylin for 30 s, then dehydrated, cleared, and mounted with neutral resin. Images were captured under a light microscope.

### 2.16. Statistical Analysis

All of the experiments were conducted at least three times. Data depicted in graphs represented the means ± SEM for each group. Student’s *t*-test was employed for comparisons between two groups and one-way analysis of variance (ANOVA), followed by Tukey’s post-test for multiple comparisons, was used for groups of three or more. The statistically significant difference between mean values was marked in each graph. *p* < 0.05 is considered significant. The statistical analyses were conducted by using GraphPad Prism 8.0 (GraphPad Software, San Diego, CA, USA).

## 3. Results

### 3.1. UA Stimulation Upregulates USP11 Expression and Promotes Apoptosis in TECs

In our in vitro experiments, we stimulated HK-2 cells with UA and found that UA upregulated USP11 expression in HK-2 in both dose-dependent and time-dependent manners. Among the conditions tested, stimulation with 800 μM UA for 36 h yielded the most pronounced effect and was therefore selected as the optimal condition for subsequent cellular assays (Supplementary Figure S1A–D). Consistent with these observations, immunoblotting results confirmed that UA stimulation under this condition also induced apoptosis in HK-2, as evidenced by upregulation of the pro-apoptotic protein Bax, downregulation of the anti-apoptotic protein Bcl-2, and activation of both caspase-9 and caspase-3 (Supplementary Figure S1E–H). Collectively, these results indicate that USP11 expression is upregulated in HK-2 upon UA stimulation, accompanied by enhanced apoptosis.

### 3.2. Genetic or Pharmacological Inhibition of USP11 Markedly Reduces Apoptosis in UA-Stimulated HK-2 Cells

**Figure 1:**
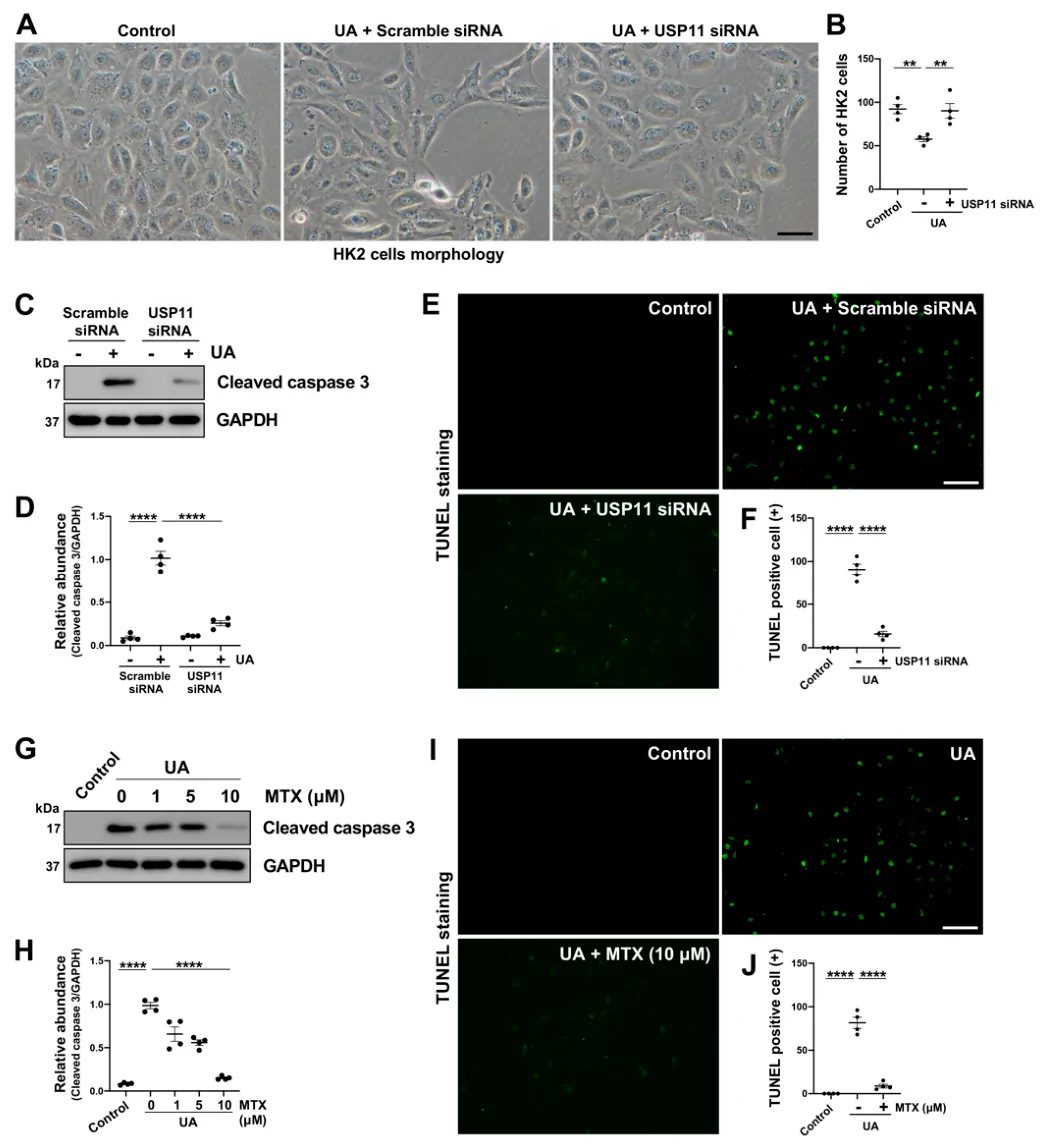
Genetic or pharmacological inhibition of USP11 markedly reduces apoptosis in UA-stimulated HK-2 cells. (**A**) Representative microscopic images of HK-2 cells with USP11 siRNA transfection under UA stimulation. (**B**) The number of viable cells was counted from the captured images. (**C**,**G**) Cell lysates were subjected to immunoblot analysis with antibodies against Cleaved caspase 3 and GAPDH. (**D**,**H**) Expression levels of Cleaved caspase 3 were quantified by densitometry and normalized with GAPDH. (**E**,**I**) Representative fluorescence images of TUNEL staining (green) in HK-2 cells. (**F**,**J**) Quantification of TUNEL-positive cells. Data were expressed as mean ± SEM (*n* = 4 for each group). ** *p* < 0.01, **** *p* < 0.0001. Scale bars = 50 μm (**A**) and 100 μm (**E**,**I**).

To suppress USP11 expression, two independent siRNAs targeting USP11 (USP11 siRNA #1 and USP11 siRNA #2) were designed and individually transfected into HK-2 cells. As shown in Supplementary Figure S2A,B, immunoblotting analysis revealed that both siRNAs markedly reduced USP11 protein levels compared with the negative control. Notably, USP11 siRNA #2 exhibited a higher knockdown efficiency than USP11 siRNA #1. Therefore, USP11 siRNA #2 was selected for subsequent functional experiments to deplete endogenous USP11 at the gene level. Following transfection, the reduced cell number observed in UA-stimulated HK-2 cells was significantly restored (Figure 1A,B). Meanwhile, knockdown of USP11 by siRNA transfection also decreased the expression level of cleaved caspase-3 (Figure 1C,D). TUNEL staining further revealed that UA stimulation alone led to a marked increase in the number of TUNEL-positive HK-2 cells, whereas transfection with USP11 siRNA reduced the number of these positive cells, indicating suppression of HK-2 apoptosis (Figure 1E,F). Furthermore, treatment with MTX, an USP11-specific inhibitor, also exerted a protective effect by reducing the occurrence of apoptosis in HK-2 cells (Figure 1G–J). Taken together, these findings indicate that genetic or pharmacological inhibition of USP11 effectively protects against UA-induced apoptosis in HK-2 cells.

### 3.3. Deubiquitination of p53 by USP11 Stabilizes the Protein and Blocks Its Proteasome-Dependent Degradation

**Figure 2:**
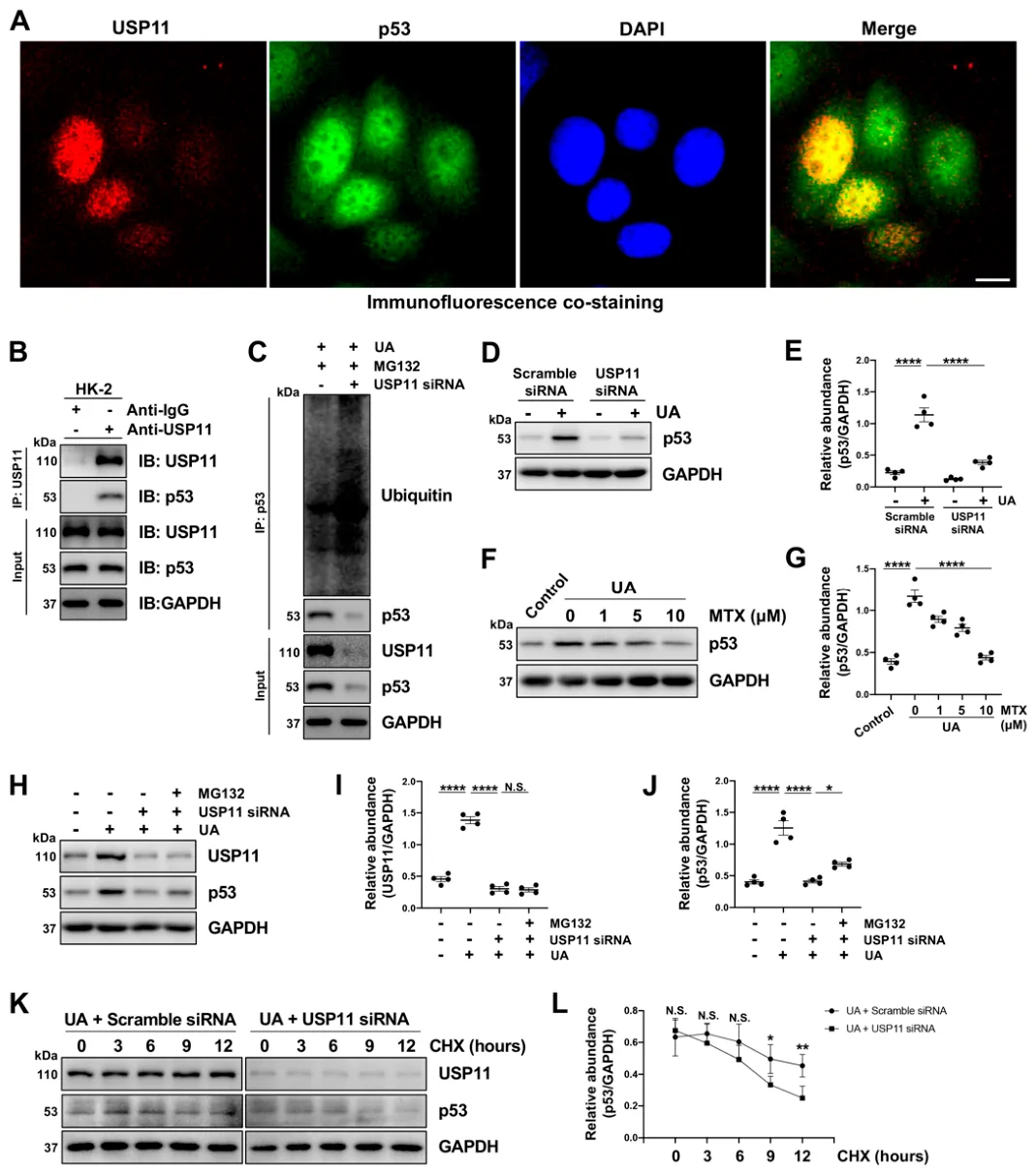
USP11-mediated deubiquitination stabilizes p53 by preventing its proteasomal degradation. (**A**) Representative immunofluorescence images showing USP11 (red), p53 (green), and DAPI (blue) staining. Merged images reveal the colocalization of USP11 and p53 (yellow signals in merge). (**B**) Cell lysates were immunoprecipitated with anti-USP11 antibody or control IgG. The immunoprecipitates and input control were then subjected to immunoblot analysis with antibodies against USP11, p53 and GAPDH. (**C**) HK-2 cells were transfected with USP11 siRNA or negative control siRNA, treated with MG132 to block proteasome-dependent degradation, and stimulated with UA. Cell lysates were immunoprecipitated with anti-p53 antibody. The immunoprecipitates and input control were then subjected to immunoblot analysis with antibodies against Ubiquitin, USP11, p53 and GAPDH. (**D**,**F**) Cell lysates were subjected to immunoblot analysis with antibodies against p53 and GAPDH. (**E**,**G**) Expression levels of p53 were quantified by densitometry and normalized with GAPDH. HK-2 cells were treated with MG132 (proteasome inhibitor) or CHX (protein synthesis inhibitor) under indicated conditions. (**H**,**K**) Cell lysates were subjected to immunoblot analysis with antibodies against USP11, p53 and GAPDH. (**I**,**J**,**L**) Expression levels of USP11, p53 were quantified by densitometry and normalized with GAPDH. Data were expressed as mean ± SEM (*n* = 4 for each group). N.S., no significant difference, * *p* < 0.05, ** *p* < 0.01, **** *p* < 0.0001. Scale bars = 10 μm (**A**).

We next sought to determine whether USP11 promotes TEC apoptosis by regulating p53. Immunofluorescence co-staining revealed colocalization of USP11 and p53, indicating an association between the two proteins (Figure 2A). Co-IP experiments further demonstrated a direct protein–protein interaction between USP11 and p53. Immunoprecipitation of USP11 with its specific antibody successfully pulled down p53 protein, as detected by immunoblotting. In contrast, the IgG control group showed no detectable p53 signal, confirming the specificity of the interaction and ruling out non-specific antibody binding (Figure 2B). Under MG132 treatment to block proteasomal degradation, ubiquitination assays showed that USP11 reduced the ubiquitination level of p53, whereas knockdown of USP11 by siRNA transfection increased p53 ubiquitination (Figure 2C). Consistently, in the presence of MTX, pharmacological inhibition of USP11 led to a pronounced increase in the ubiquitination level of p53, accompanied by a marked reduction in p53 protein abundance, suggesting that MTX promotes p53 degradation via enhanced ubiquitination (Supplementary Figure S3). Additionally, both genetic and pharmacological inhibition of USP11 confirmed that USP11 indeed regulated p53 protein expression. Immunoblotting analyses revealed that knockdown of USP11 by siRNA or inhibition of USP11 activity with graded doses of MTX both led to a marked reduction in p53 protein levels, as compared to cells treated with UA alone (Figure 2D–G). Further mechanistic investigations revealed that, under UA stimulation, treatment with MG132, a proteasome inhibitor, partially restored p53 expression levels (Figure 2H–J). CHX chase assays indicated changes in the half-life of p53 under USP11 siRNA transfection, suggesting that USP11 affected p53 protein stability (degradation rate) rather than its transcription or translation (Figure 2K,L). Taken together, these results demonstrate that USP11 deubiquitinates and stabilizes p53, thereby protecting it from ubiquitin-proteasome-dependent degradation.

### 3.4. USP11 Mediates the Mitochondrial Apoptotic Pathway in HK-2 by Regulating p53

**Figure 3:**
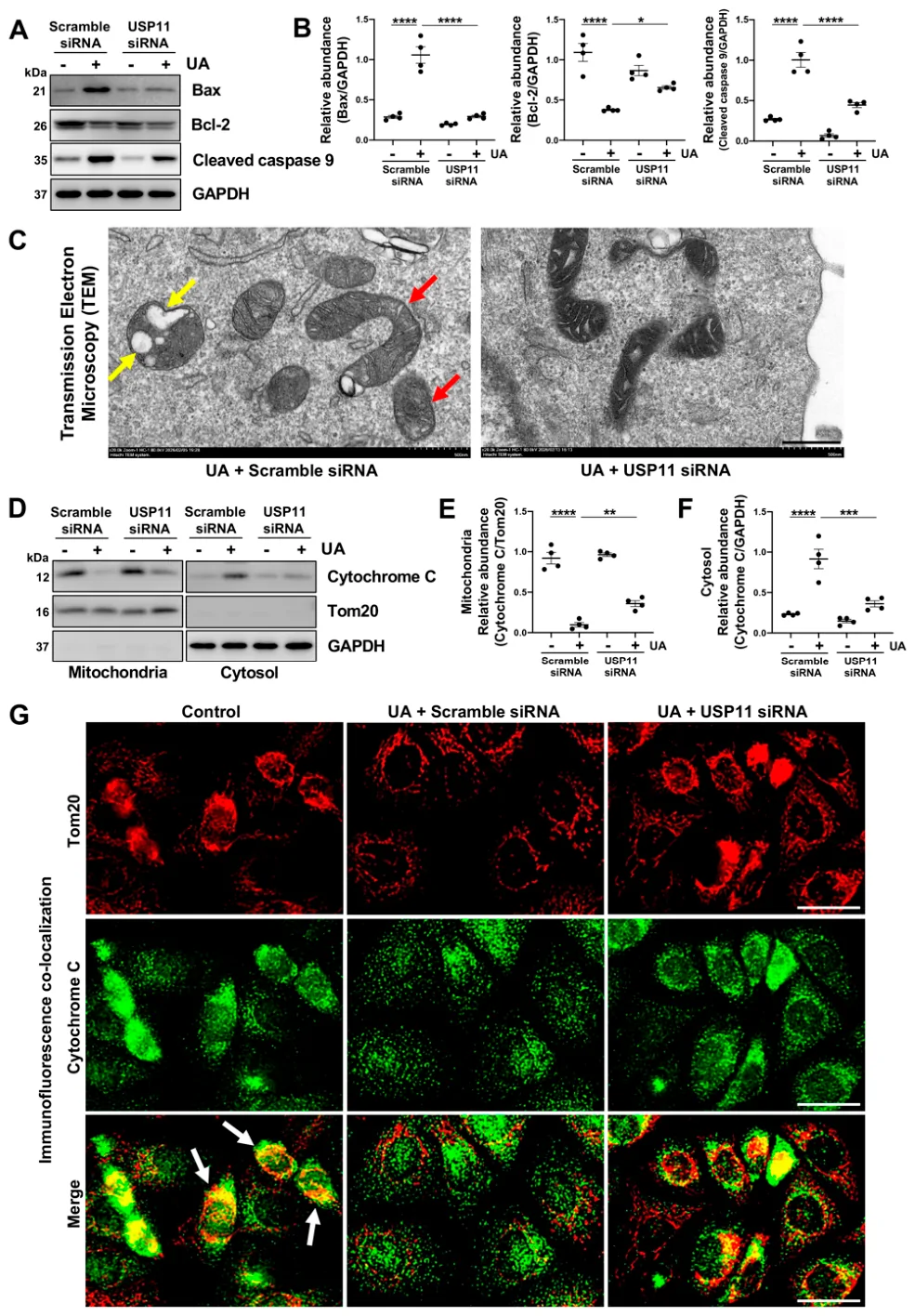
USP11 mediates the mitochondrial apoptotic pathway and regulates cytochrome c release from mitochondria into the cytosol in HK-2 cells. (**A**) Cell lysates were subjected to immunoblot analysis with antibodies against Bax, Bcl-2, Cleaved caspase 9 and GAPDH. (**B**) Expression levels of Bax, Bcl-2, Cleaved caspase 9 were quantified by densitometry and normalized with GAPDH. (**C**) Representative TEM images of HK-2 cells under indicated conditions. Red arrows indicate damaged mitochondria, characterized by swelling and sparse, fragmented, or even absent cristae. Yellow arrows point to vacuolization within mitochondria. Then, we isolated the mitochondrial and cytoplasmic fractions of HK-2 cells and verified the fraction purity using Tom20 (a mitochondrial marker) and GAPDH (a cytoplasmic marker). (**D**) Mitochondrial and cytoplasmic fractions were subjected to immunoblot analysis with antibodies against Cytochrome C, Tom20 and GAPDH. (**E**,**F**) Expression levels of Cytochrome C were quantified by densitometry and normalized with Tom20 and GAPDH, respectively. (**G**) Representative immunofluorescence images showing Tom20 (red) and Cytochrome C (green) staining. Merged images reveal the colocalization of Tom20 and Cytochrome C (yellow signals in merge, as pointed by white arrows). Data were expressed as mean ± SEM (*n* = 4 for each group). * *p* < 0.05, ** *p* < 0.01, *** *p* < 0.001, **** *p* < 0.0001. Scale bars = 500 nm (**C**) and 25 μm (**G**).

Immunoblotting results showed that transfection with USP11 siRNA reduced Bax expression while restoring Bcl-2 expression, thereby correcting the imbalance in the Bax/Bcl-2 ratio induced by UA stimulation (Figure 3A,B). TEM of HK-2 cells revealed that UA stimulation disrupted mitochondrial architecture, as evidenced by mitochondrial swelling, sparse, fragmented, or even absent cristae, and vacuolization. In contrast, cells transfected with USP11 siRNA exhibited relatively normal mitochondrial morphology. These findings indicate that USP11 contributes to mitochondrial structural damage and increased MOMP (Figure 3C). Increased MOMP promotes the release of cytochrome c from mitochondria into the cytosol [18,19].

Therefore, we isolated the mitochondrial and cytoplasmic fractions of HK-2 cells and verified the fraction purity using Tom20 (a mitochondrial marker) and GAPDH (a cytoplasmic marker). Under normal conditions, cytochrome c was predominantly localized in the mitochondria. Upon UA stimulation, cytochrome c levels in the mitochondrial fraction significantly decreased, whereas its levels in the cytoplasmic fraction markedly increased, indicating that cytochrome c had been released from the mitochondria into the cytosol during this process. However, upon USP11 inhibition, cytoplasmic cytochrome c levels decreased, whereas mitochondrial cytochrome c levels conversely increased. These reciprocal changes indicate that USP11 inhibition effectively reduces the release of cytochrome c from the mitochondria into the cytosol, thereby blocking the onset of apoptosis (Figure 3D–F). Furthermore, this trend was more intuitively visualized by immunofluorescence staining. In control HK-2 cells, cytochrome c and Tom20 exhibited substantial co-localization, indicating that cytochrome c was primarily localized within the mitochondria. Upon UA stimulation, cytochrome c displayed a diffuse distribution pattern and showed reduced overlap with Tom20, suggesting that cytochrome c was predominantly localized in the cytosol under this condition (Figure 3G). Subsequently, cytochrome c activated caspase 9, thereby initiating the apoptotic cascade (Figure 3A,B).

**Figure 4:**
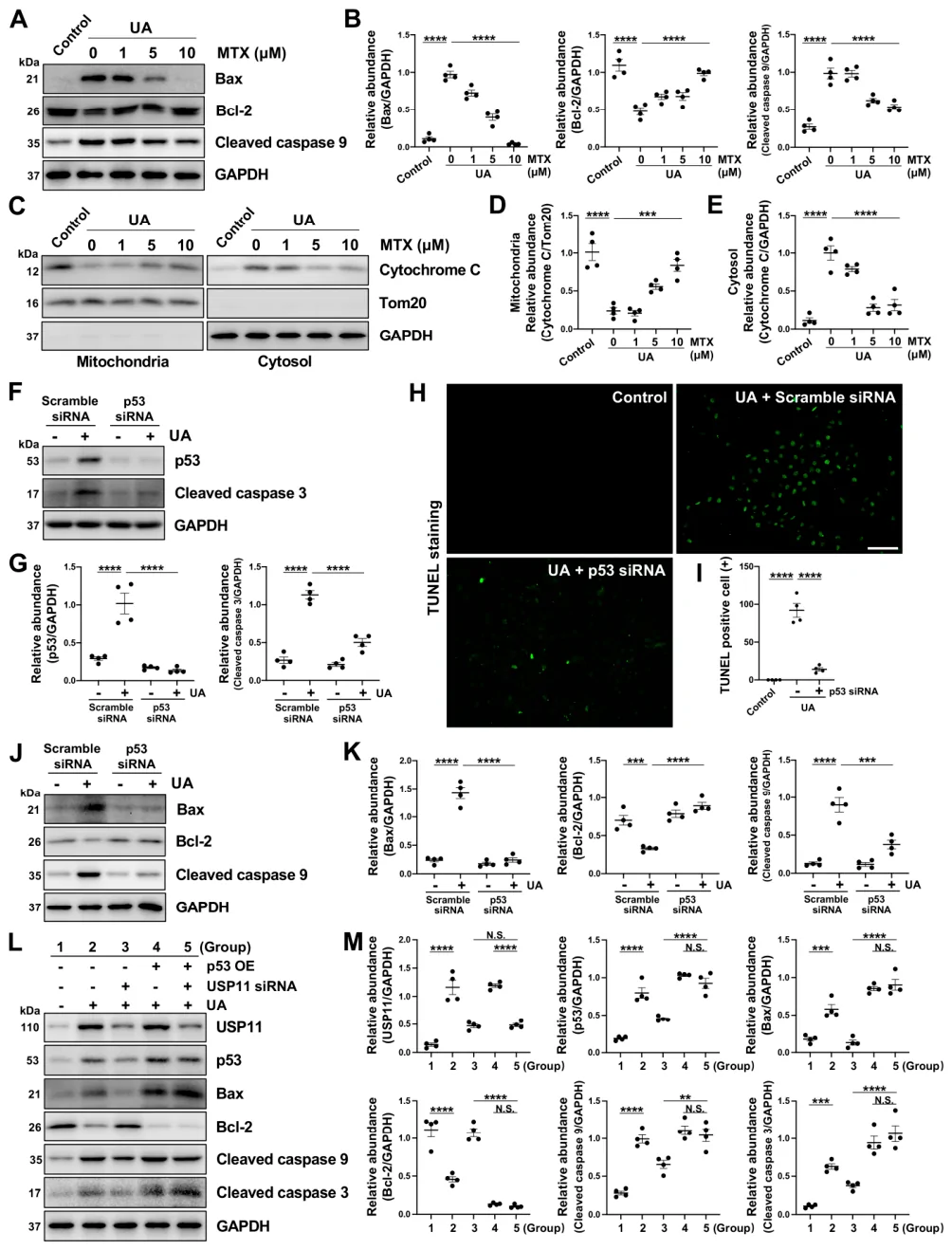
USP11 mediates the mitochondrial apoptotic pathway in HK-2 by regulating p53. (**A**) Cell lysates were subjected to immunoblot analysis with antibodies against Bax, Bcl-2, Cleaved caspase 9 and GAPDH. (**B**) Expression levels of Bax, Bcl-2, Cleaved caspase 9 were quantified by densitometry and normalized with GAPDH. (**C**) Mitochondrial and cytoplasmic fractions were subjected to immunoblot analysis with antibodies against Cytochrome C, Tom20 and GAPDH. (**D**,**E**) Expression levels of Cytochrome C were quantified by densitometry and normalized with Tom20 and GAPDH, respectively. To further validate the involvement of p53 in the mitochondrial apoptotic cascade, we transfected HK-2 cells with p53-specific siRNA. (**F**) Cell lysates were subjected to immunoblot analysis with antibodies against p53, Cleaved caspase 3 and GAPDH. (**G**) Expression levels of p53, and Cleaved caspase 3 were quantified by densitometry and normalized with GAPDH. (**H**) Representative fluorescence images of TUNEL staining (green) in HK-2 cells. (**I**) Quantification of TUNEL-positive cells. (**J**) Cell lysates were subjected to immunoblot analysis with antibodies against Bax, Bcl-2, Cleaved caspase 9 and GAPDH. (**K**) Expression levels of Bax, Bcl-2, and Cleaved caspase 9 were quantified by densitometry and normalized with GAPDH. (**L**) For rescue assays, UA-stimulated HK-2 cells were transfected with USP11 siRNA and/or p53 pcDNA3.1 plasmid (p53 OE). Cell lysates were subjected to immunoblot analysis with antibodies against USP11, p53, Bax, Bcl-2, Cleaved caspase 9, Cleaved caspase 3 and GAPDH. (**M**) Expression levels of USP11, p53, Bax, Bcl-2, Cleaved caspase 9, and Cleaved caspase 3 were quantified by densitometry and normalized with GAPDH. Data were expressed as mean ± SEM (*n* = 4 for each group). N.S., no significant difference, ** *p* < 0.01, *** *p* < 0.001, **** *p* < 0.0001. Scale bars = 100 μm (**H**).

To validate the specificity of USP11-mediated regulation of the p53 signaling pathway, two independent siRNAs targeting distinct sequences of USP11 (#1 and #2) were individually transfected into HK-2 cells. As shown in Supplementary Figure S2C,D, both USP11 siRNAs consistently downregulated p53 protein expression, suppressed Bax levels compared with the negative control, and inhibited the activation of caspase 9 and caspase 3. Given that two sequence-independent siRNAs produced highly concordant effects on p53, Bax, and downstream caspase activation, these results demonstrate that the observed regulatory effects are specifically attributed to USP11 depletion rather than off-target artifacts. Similarly, pharmacological inhibition of USP11 using MTX produced similar protective effects against apoptosis in HK-2 cells. Treatment with MTX dose-dependently reduced Bax expression, while concurrently restoring Bcl-2 levels. Consequently, this suppressed the release of cytochrome c from the mitochondria into the cytosol and inhibited the activation of caspase-9 (Figure 4A–E). In contrast, overexpression of USP11 exerted opposite effects, leading to upregulated p53 expression and increased apoptosis, as evidenced by enhanced cleavage of caspase 9 and caspase 3 (Supplementary Figure S4).

To further demonstrate that USP11 promotes apoptosis through p53, we transfected HK-2 cells with p53-specific siRNA and examined downstream apoptotic changes following p53 inhibition (Figure 4F–K). The results confirmed that knockdown of p53 significantly reduced the number of TUNEL-positive cells, downregulated Bax expression, restored Bcl-2 expression, and subsequently suppressed the activation of both caspase 9 and caspase 3. Rescue experiments revealed that p53 overexpression reversed the anti-apoptotic effect of USP11 knockdown, whereas USP11 knockdown did not affect p53 overexpression-induced apoptosis (Figure 4L,M). These findings indicate that USP11 regulates the mitochondrial apoptotic pathway in HK-2 cells specifically through p53, which functions downstream of USP11 in this process.

### 3.5. Genetic Knockout of USP11 Attenuates Apoptosis and Ameliorates Kidney Injury in a Mouse Model of Hyperuricemic Nephropathy

**Figure 5:**
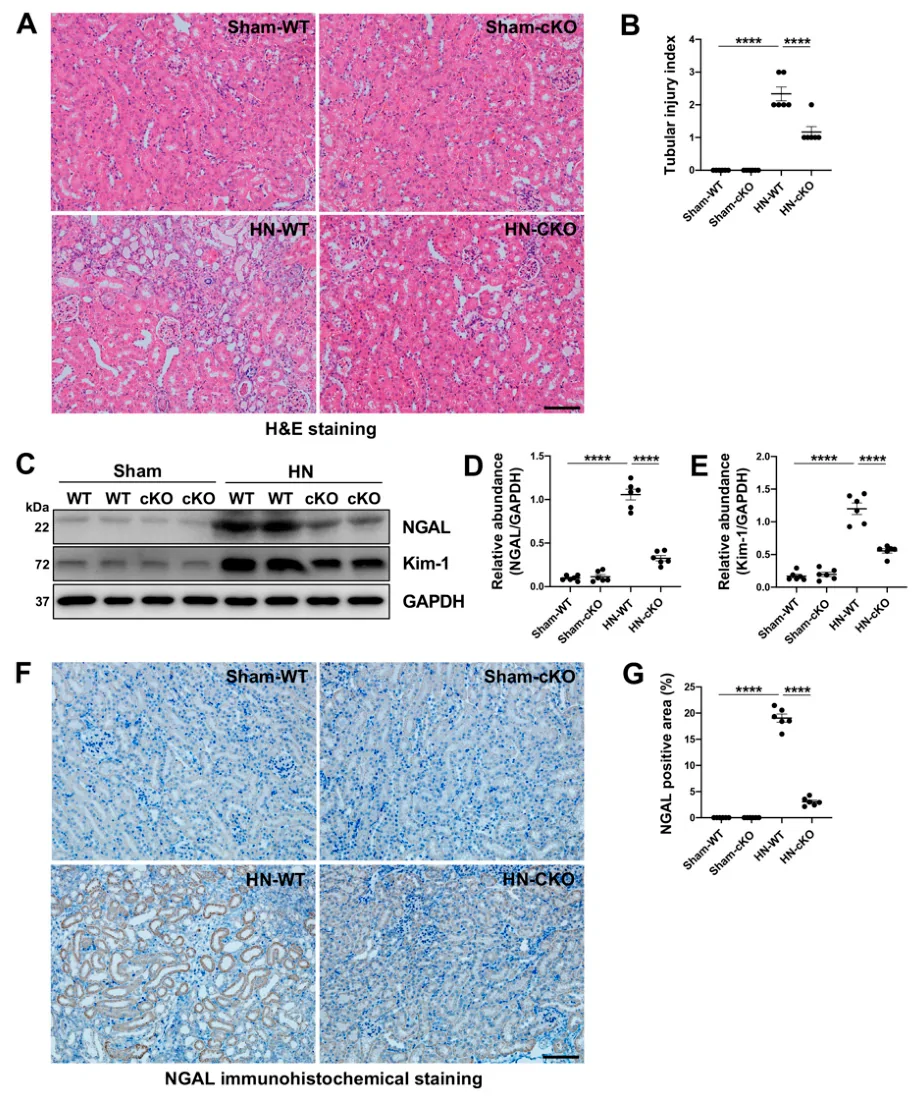
Genetic knockout of USP11 attenuates renal tubular injury in a mouse model of hyperuricemic nephropathy. To further elucidate the role of USP11 and its regulation of p53-mediated apoptosis in the progression of CKD, we generated USP11-conditional knockout (cKO) mouse in renal tubular epithelial cells (using Cdh16-Cre) and established a HN model, a well-characterized mouse model of CKD injury. (**A**) Representative hematoxylin and eosin (H&E) staining of kidney sections from each indicated group. (**B**) Quantification of tubular injury index. (**C**) Kidney tissues were subjected to immunoblot analysis with antibodies against NGAL, Kim-1 and GAPDH. (**D**,**E**) Expression levels of NGAL, Kim-1 were quantified by densitometry and normalized with GAPDH. (**F**) Representative immunohistochemical staining of NGAL in kidney sections from each indicated group. (**G**) Quantitative analysis of NGAL-positive area. Data were expressed as mean ± SEM (*n* = 6 for each group). **** *p* < 0.0001. Scale bars = 100 μm (**A**,**F**).

To further elucidate the role of USP11 and its regulation of p53-mediated apoptosis in the progression of CKD, we generated a USP11-conditional knockout (cKO) mouse in renal tubular epithelial cells (using Cdh16-Cre) and established a HN model, a well-characterized mouse model of CKD injury. H&E staining revealed that mice in the HN model group exhibited severe tubular injury, whereas tubular damage was substantially alleviated upon genetic knockout of USP11 (Figure 5A). Consistently, the tubular injury index was significantly lower in HN-cKO mice compared with the HN model group (Figure 5B). Immunoblotting results showed that levels of NGAL and Kim-1, two markers of kidney injury, were elevated in the HN model group relative to control mice, while their expression levels were reduced in USP11-cKO mice following HN (Figure 5C–E). Furthermore, immunohistochemical staining for NGAL confirmed that conditional knockout of USP11 ameliorated the extent of kidney injury in HN mice (Figure 5F,G).

**Figure 6:**
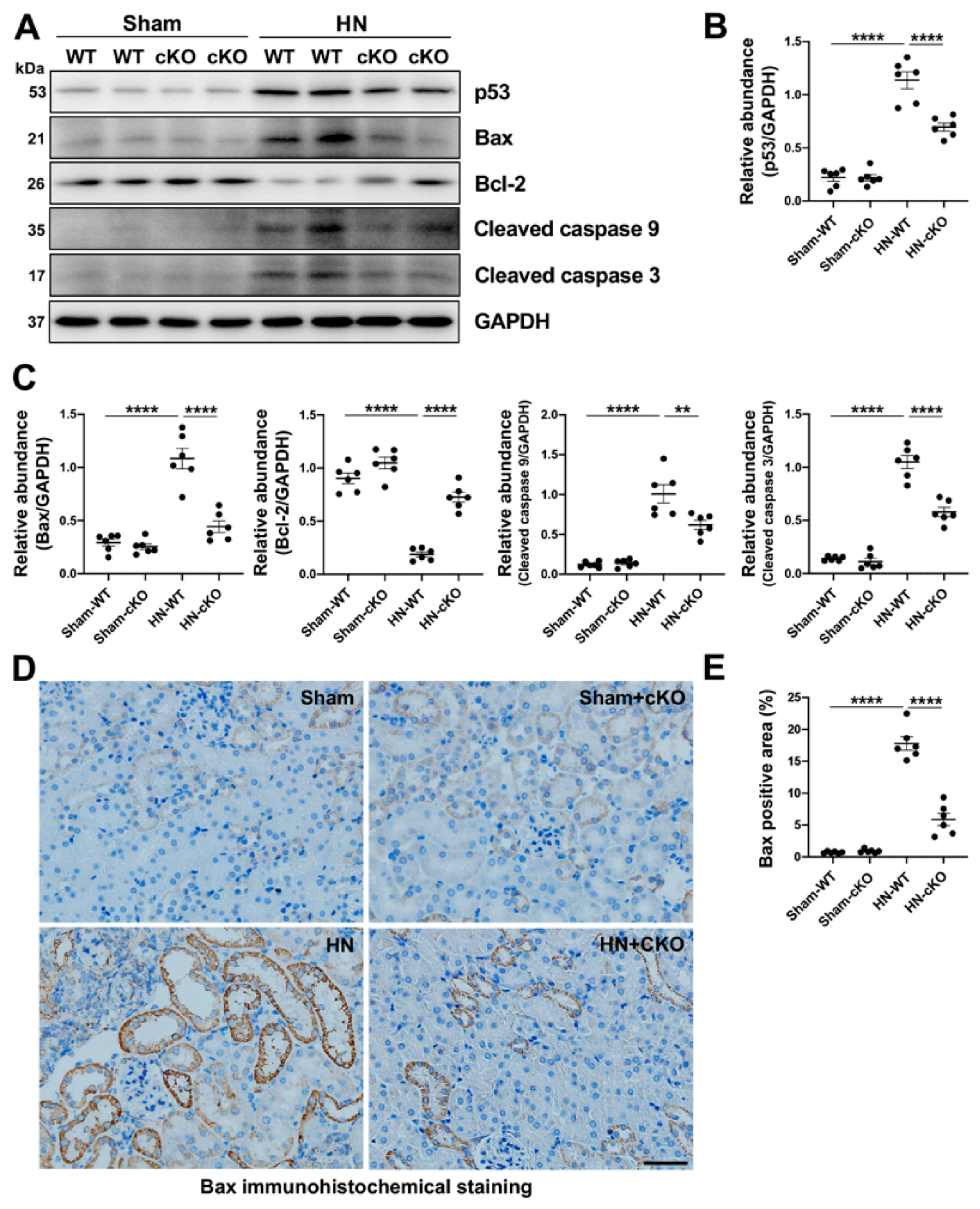
Genetic knockout of USP11 suppresses apoptosis in a mouse model of hyperuricemic nephropathy. (**A**) Kidney tissues were subjected to immunoblot analysis with antibodies against p53, Bax, Bcl-2, Cleaved caspase 9, Cleaved caspase 3 and GAPDH. (**B**,**C**) Expression levels of p53, Bax, Bcl-2, Cleaved caspase 9, and Cleaved caspase 3 were quantified by densitometry and normalized with GAPDH. (**D**) Representative immunohistochemical staining of Bax in kidney sections from each indicated group. (**E**) Quantitative analysis of Bax-positive area. Data were expressed as mean ± SEM (*n* = 6 for each group). ** *p* < 0.01, **** *p* < 0.0001. Scale bars = 50 μm (**D**).

Furthermore, in vivo experiments demonstrated that p53 was highly expressed in the kidneys of HN mice, whereas USP11 genetic knockout significantly reduced renal p53 expression (Figure 6A,B). Additionally, deletion of USP11 downregulated Bax expression and, conversely, upregulated the protective protein Bcl-2 (Figure 6A,C). Regarding the caspase family, the expression levels of cleaved caspase 9 and cleaved caspase 3 in the kidneys of HN-cKO mice were substantially decreased compared with those in the HN model group, indicating that USP11 genetic knockout effectively suppressed apoptosis (Figure 6A,C). Finally, immunohistochemical staining for Bax in mouse kidney revealed that USP11 genetic knockout reduced the area of Bax-positive staining (Figure 6D,E). Collectively, these findings indicate that genetic knockout of USP11 protects against apoptosis and alleviates renal injury in a mouse model of HN.

### 3.6. Pharmacological Inhibition of USP11 Effectively Blocks the p53-Mediated Apoptotic Pathway in the HN Model

**Figure 7:**
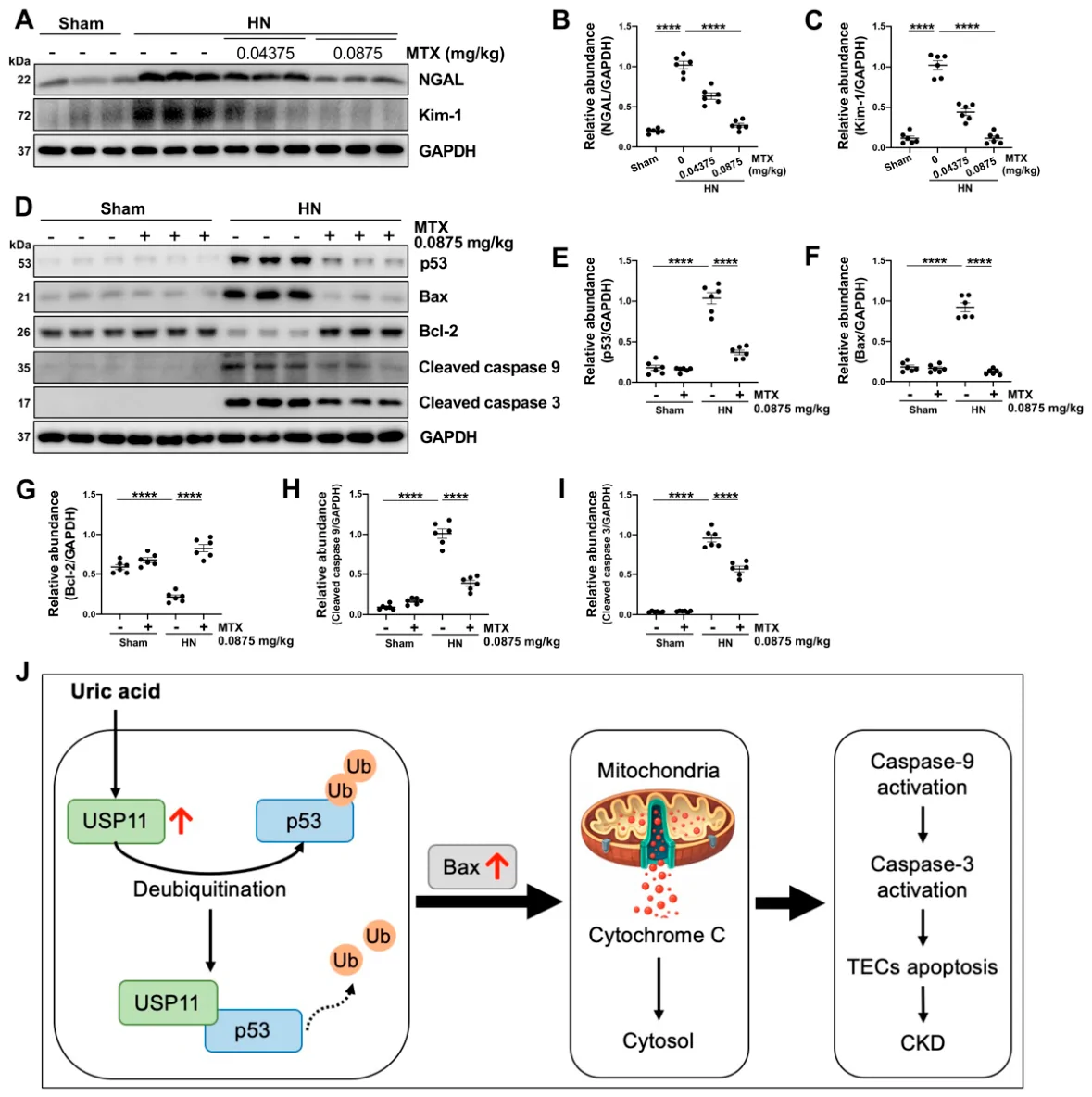
Pharmacological inhibition of USP11 effectively blocks the p53-mediated apoptotic pathway in the hyperuricemic nephropathy mouse model. In addition to genetic knockout, we also used the pharmacological inhibitor MTX to suppress USP11 expression at doses of 0.04375 mg/kg and 0.0875 mg/kg. (**A**) Kidney tissues were subjected to immunoblot analysis with antibodies against NGAL, Kim-1 and GAPDH. (**B**,**C**) Expression levels of NGAL, Kim-1 were quantified by densitometry and normalized with GAPDH. (**D**) Kidney tissues were subjected to immunoblot analysis with antibodies against p53, Bax, Bcl-2, Cleaved caspase 9, Cleaved caspase 3 and GAPDH. (**E**–**I**) Expression levels of p53, Bax, Bcl-2, Cleaved caspase 9, and Cleaved caspase 3 were quantified by densitometry and normalized with GAPDH. (**J**) Schematic diagram illustrating the proposed mechanism by which USP11 regulates tubular epithelial cell apoptosis in CKD progression. Data were expressed as mean ± SEM (*n* = 6 for each group). **** *p* < 0.0001.

In addition to genetic knockout, we also used the pharmacological inhibitor MTX to suppress USP11 expression. The results showed that MTX reduced the expression levels of NGAL and Kim-1 in HN mice in a dose-dependent manner, with the optimal inhibitory effect observed at a dose of 0.0875 mg/kg (Figure 7A–C). Similarly, pharmacological inhibition of USP11 with MTX also downregulated renal p53 expression and effectively suppressed p53-mediated apoptosis, as evidenced by downregulation of Bax, upregulation of Bcl-2, and reduced the activation levels of caspase 9 and caspase 3 (Figure 7D–I). Taken together, our results demonstrate that MTX-mediated inhibition of USP11 effectively protects the kidneys from injury. Considering that MTX is already an FDA-approved agent with a well-established safety profile, our findings raise the possibility of repurposing this drug for the treatment of CKD, particularly in patients with HN. This offers a promising and rapidly translatable therapeutic avenue beyond its conventional use in cancer therapy.

## 4. Discussion

Although previous studies have confirmed that USP11 regulates partial EMT and pro-fibrotic responses in TECs during the progression of CKD [16,17], its role in apoptosis of TECs has not yet been reported. In the present study, UA stimulation was observed to upregulate USP11 expression in TECs in both dose- and time-dependent patterns, accompanied by the induction of apoptosis. Targeted inhibition of USP11, either pharmacologically or genetically, markedly suppressed the occurrence of apoptosis, whereas USP11 overexpression exerted the opposite effect, significantly enhancing apoptotic cell death. Mechanistically, USP11 was found to directly interact with p53. As a deubiquitinating enzyme, USP11 deubiquitinated p53, thereby shielding it from proteasomal degradation and stabilizing the p53 protein. Upon stabilization, p53 became activated and functioned as a transcription factor to upregulate the pro-apoptotic protein Bax while downregulating the anti-apoptotic protein Bcl-2. This, in turn, enhanced MOMP, promoted cytochrome c release into the cytoplasm, and activated caspase 9 and caspase 3, ultimately driving TECs apoptosis and contributing to kidney injury (Figure 7J). Collectively, our findings reaffirm the critical role of USP11 in CKD progression and highlight a promising therapeutic strategy by targeting USP11 in CKD patients.

Our co-IP experiments demonstrated a direct protein–protein interaction between USP11 and p53. Ubiquitination assays showed that USP11 reduced the ubiquitin levels on p53, whereas both MTX treatment and siRNA-mediated knockdown of USP11 increased p53 ubiquitination. MG132 and CHX assays confirmed that USP11 protected p53 from proteasome-dependent degradation, thereby maintaining p53 protein stability and facilitating its downstream biological functions. Together, these results provide strong evidence that USP11 exerts its pro-apoptotic effect in TECs via p53. Beyond our findings, other studies have also reported a direct functional interaction between USP11 and p53 in the context of other diseases. Zhang et al. reported that USP11 acts as a pro-inflammatory mediator by enhancing p53 stability by deubiquitination and inhibiting KLF2 in intracerebral hemorrhage [20]. Ke et al. reported that USP11 forms specific complexes with p53 and stabilizes p53 by protecting it from ubiquitin-dependent degradation in response to DNA damage stress [21]. Taken together, these findings, along with our own, consistently establish USP11 as a context-independent deubiquitinating enzyme for p53.

In contrast to previous studies that primarily characterized the USP11-p53 interaction in cancer cell lines and focused on cell-cycle regulation or DNA damage responses, our study reveals a previously unrecognized pathophysiological role for this signaling axis in TECs apoptosis during CKD progression. Specifically, we demonstrate that USP11-mediated stabilization of p53 triggers a downstream mitochondrial apoptotic program characterized by MOMP, cytochrome c release, activation of the caspase family, and subsequent tubular epithelial cell apoptosis. This disease-specific downstream mechanism has not been previously described for the USP11-p53 axis. Importantly, p53 functions as a critical mechanistic mediator linking USP11 to renal tubular epithelial injury and subsequent renal fibrosis. Therefore, rather than merely confirming the previously reported deubiquitination of p53 by USP11, our study extends these findings by defining the pathological significance and downstream consequences of this interaction in CKD, thereby expanding its biological relevance and identifying USP11 as a potential therapeutic target for renal fibrosis.

Apoptosis of TECs represents a critical initiating event that drives the progression of CKD [5,6]. In the present study, we focused on p53-mediated mitochondrial pathway of apoptosis during CKD. Through mitochondrial isolation and immunofluorescence co-localization of cytochrome c with Tom20, we demonstrated that upon apoptosis induction, cytochrome c is released from the mitochondria into the cytoplasm, thereby serving as an irreversible trigger of the apoptotic process. Beyond apoptosis, other forms of programmed cell death (PCD) in TECs have also been implicated in the pathogenesis of kidney injury [5]. In diabetic kidney disease (DKD), high glucose and fatty acids aggravate renal injury by inducing RIPK1/RIPK3-dependent necroptosis in TECs. Pharmacological inhibition of RIPK1 effectively blocks this necroptotic pathway, thereby alleviating necroinflammation and renal damage [22]. Similarly, in DKD, high glucose conditions have also been shown to induce pyroptosis in TECs, accompanied by inflammasome activation and subsequent release of inflammatory cytokines [23]. Notably, the expression levels of inflammation-related protein NOD-like receptor protein 3 (NLRP3) and pyroptosis key protein gasdermin D (GSDMD) in kidney tissues from patients with DKD were elevated compared with those observed in control subjects [23]. One of the key distinctions between apoptosis and pyroptosis lies in their differential dependence on caspase 3 as the central executioner. In our study, USP11-p53 axis inhibition significantly reduced caspase 3 activation, and TEM revealed no typical pyroptotic features such as cell swelling, membrane rupture, or cytoplasmic leakage. These findings indicate that the USP11-p53 pathway primarily drives apoptotic cell death rather than pyroptosis in TECs under our experimental conditions. Furthermore, ferroptosis in TECs has been established as a contributing mechanism in diverse types of kidney injury, such as DKD [24], unilateral ureteral obstruction (UUO)-induced renal fibrosis [25], acute kidney injury (AKI) [26], and the AKI-to-CKD transition [27]. In summary, multiple PCD pathways of TECs can ultimately contribute to the progression of kidney injury. Whether USP11 also plays a role in other PCD pathways remains to be clarified by further investigations.

In addition to the previously reported deubiquitination substrates of USP11 in CKD progression, including EGFR [17], TGFβRII [16] and KLF4 [28], our study identifies p53 as a novel substrate of USP11 in TEC apoptosis. However, given the versatile enzymatic activity of USP11, the full repertoire of its substrates in the context of kidney injury likely extends beyond these identified molecules. A recent research demonstrates that USP11 exacerbates radiation-induced pneumonitis (RIP) by activating the OTUD5-STING signaling axis to promote endothelial cell inflammatory responses [29]. The kidney is notably rich in vasculature, with microvascular endothelial cells densely distributed around the glomeruli and tubules. Once inflammatory responses are triggered in these endothelial cells, their activation leads to the expression of adhesion molecules such as intercellular adhesion molecule-1 (ICAM-1) and vascular cell adhesion molecule-1 (VCAM-1), which in turn recruit inflammatory cells, including macrophages and T cells, into the kidney [30,31]. These infiltrating cells release pro-fibrotic cytokines such as TGF-β and IL-6, thereby directly promoting tubulointerstitial fibrosis, a cornerstone pathological process driving the progression of CKD to ESRD [32,33]. Given this, it is highly plausible that USP11 is also involved in endothelial cell inflammatory responses during CKD progression. Whether and how these candidate substrates are regulated by USP11 in the injured kidney warrants further investigation.

MTX (a small-molecule inhibitor of USP11) is currently approved for clinical use primarily in two major therapeutic areas: oncology and autoimmune diseases. In cancer treatment, it is commonly used for acute leukemia, breast cancer, lymphoma, and hormone-refractory prostate cancer [34]. In the context of autoimmunity, it is an FDA-approved second-line agent for the management of progressive forms of multiple sclerosis [35,36]. In addition, multiple clinical trials involving mitoxantrone have demonstrated favorable clinical therapeutic efficacy. MTX-based regimens demonstrated clinical activity in relapsed/refractory (R/R) acute myeloid leukemia (AML), achieving measurable remission rates while maintaining manageable safety profiles [37]. Another retrospective study showed that the combination of etoposide and MTX achieved a complete remission rate of 39% in newly diagnosed AML patients who failed initial cytarabine and idarubicin therapy, demonstrating meaningful salvage efficacy [38]. In both in vitro and in vivo settings of the present study, we also employed MTX to further investigate its functional impact. Our results demonstrated that pharmacological inhibition of USP11 by MTX significantly suppressed apoptosis in cultured TECs. Moreover, in a mouse HN model of CKD, MTX treatment dose-dependently attenuated tubular cell apoptosis, as evidenced by reduced expression of the tubular injury markers NGAL and Kim-1, along with improved tubular injury scores, ultimately leading to amelioration of kidney injury. MTX therefore holds promise as a candidate therapy for clinical management of CKD, pending further validation in preclinical and translational studies.

In summary, this study reveals that USP11 promotes tubular epithelial cell apoptosis by deubiquitinating and stabilizing p53, thereby activating the mitochondrial death pathway. Targeted inhibition of USP11, either genetically or pharmacologically with MTX, effectively suppresses apoptosis of TECs and alleviates kidney injury. Collectively, our findings not only establish USP11 as a critical promoter of tubular apoptosis in CKD but also identify its inhibitor, mitoxantrone, as a promising and rapidly repurposable candidate for mitigating kidney injury, offering a new therapeutic avenue beyond its conventional indications.

## Data Availability

The data analyzed during this study are included in this published article. Additional supporting data are available from the corresponding author upon reasonable request.

## Supplementary Materials

The following supporting information can be downloaded at: https://www.mdpi.com/article/10.3390/biomedicines14081852/s1, Figure S1: Uric acid stimulation upregulates USP11 expression and promotes apoptosis in TECs; Figure S2: Knockdown of USP11 by two independent siRNAs transfection suppresses p53 expression and apoptosis in HK-2 cells; Figure S3: MTX treatment enhances p53 ubiquitination and decreases p53 protein stability in HK-2 cells; Figure S4: Overexpression of USP11 upregulates p53 expression and promotes apoptosis in HK-2 cells.
