# Supplementary material for: Ubiquitin-Specific Protease 11 Promotes Uric Acid-Induced Renal Tubular Epithelial Cell Apoptosis via Deubiquitinating P53 and Enhancing Mitochondrial Outer Membrane Permeabilization
Source: Biomedicines. 2026 Aug 18;14(8):1852. doi: 10.3390/biomedicines14081852 (PMC13509996; doi:10.3390/biomedicines14081852)
Supplement: Supplementary file 1 [file biomedicines-14-01852-s001.zip › biomedicines-4406078-supplementary.pdf]

Supplementary materials

Supplementary Figure S1-S4

Ubiquitin-Specific Protease 11 Promotes Uric Acid-Induced Renal Tubular Epithelial Cell Apoptosis via Deubiquitinating P53 and Enhancing Mitochondrial Outer Membrane Permeabilization

Yingfeng Shi[1], Jinqing Li[1], Na Liu[1*]

[1]Department of Nephrology, Shanghai East Hospital, School of Medicine, Tongji University, Shanghai 200120, China.

[*]**Correspondence and offprint requests to:** Na Liu, M.D., Ph.D., Department of Nephrology, Shanghai East Hospital, School of Medicine, Tongji University, 150 Jimo road, Pudong new district, Shanghai 200120, China.

E-mail: naliubrown@163.com

Running Title: USP11 promotes apoptosis via p53

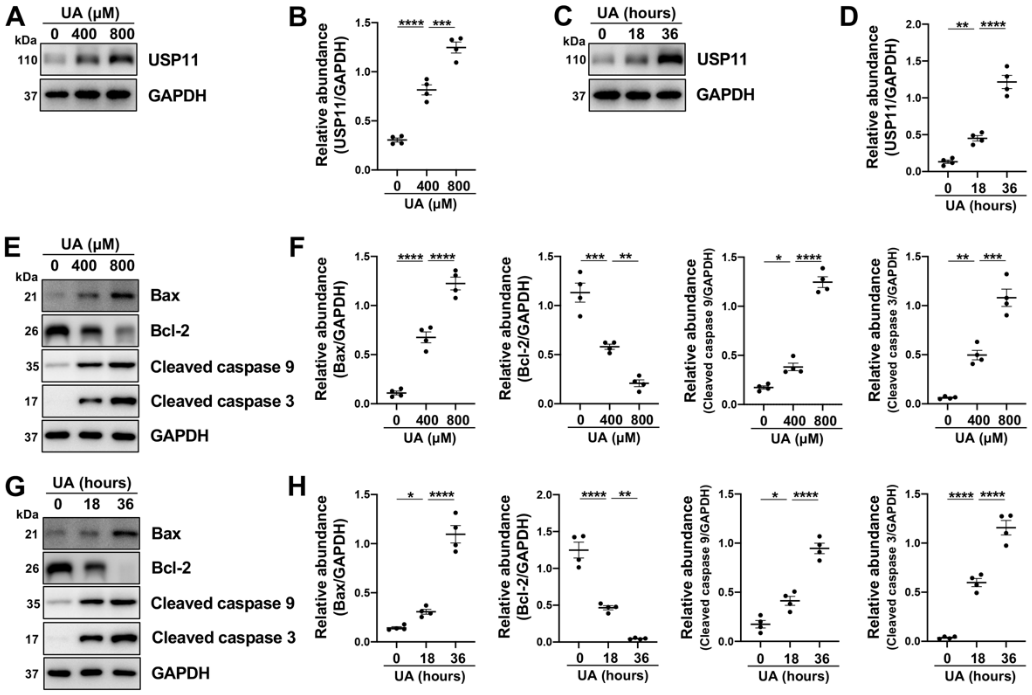

A
UA (μM)
0 400 800
kDa
110 USP11
37 GAPDH
B
Relative abundance (USP11/GAPDH)
**** ***
UA (μM)
C
UA (hours)
0 18 36
110 USP11
37 GAPDH
D
Relative abundance (USP11/GAPDH)
** ****
UA (hours)
E
UA (μM)
0 400 800
21 Bax
26 Bcl-2
35 Cleaved caspase 9
17 Cleaved caspase 3
37 GAPDH
F
Relative abundance (Bax/GAPDH)
Relative abundance (Bcl-2/GAPDH)
Relative abundance (Cleaved caspase 9/GAPDH)
Relative abundance (Cleaved caspase 3/GAPDH)
UA (μM)
G
UA (hours)
0 18 36
21 Bax
26 Bcl-2
35 Cleaved caspase 9
17 Cleaved caspase 3
37 GAPDH
H
Relative abundance (Bax/GAPDH)
Relative abundance (Bcl-2/GAPDH)
Relative abundance (Cleaved caspase 9/GAPDH)
Relative abundance (Cleaved caspase 3/GAPDH)
UA (hours)

Supplementary Figure S1. Uric acid stimulation upregulates USP11 expression and promotes apoptosis in TECs

In in vitro experiments, we stimulated HK-2 cells with UA in both dose-dependent and time-dependent manners. (A, C) Cell lysates were subjected to immunoblot analysis with antibodies against USP11 and GAPDH. (B, D) Expression levels of USP11 were quantified by densitometry and normalized with GAPDH. (E, G) Cell lysates were subjected to immunoblot analysis with antibodies against Bax, Bcl-2, Cleaved caspase 9, Cleaved caspase 3 and GAPDH. (F, H) Expression levels of Bax, Bcl-2, Cleaved caspase 9, Cleaved caspase 3 were quantified by densitometry and normalized with GAPDH. Data were expressed as mean ± SEM (*n*=4 for each group). *P<0.05, **P<0.01, ***P<0.001, ****P<0.0001.

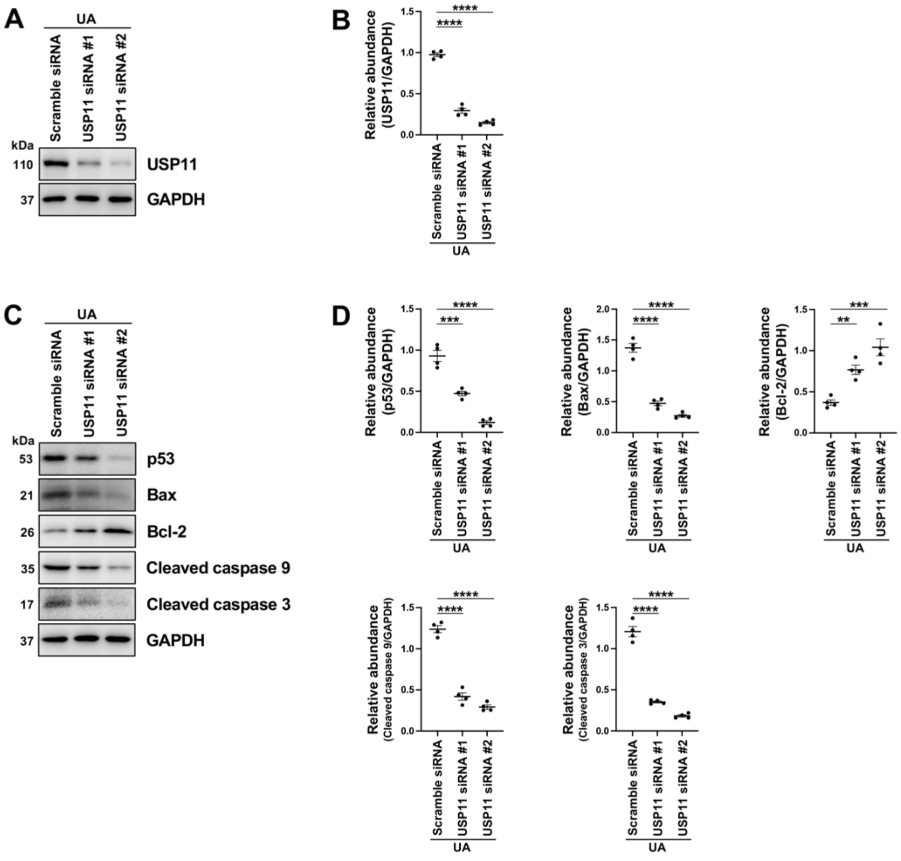
A
UA
Scramble siRNA
USP11 siRNA #1
USP11 siRNA #2
kDa
110
USP11
37
GAPDH
B
Relative abundance (USP11/GAPDH)
1.5
1.0
0.5
0.0
****
****
Scramble siRNA
USP11 siRNA #1
USP11 siRNA #2
UA
C
UA
Scramble siRNA
USP11 siRNA #1
USP11 siRNA #2
kDa
53
p53
21
Bax
26
Bcl-2
35
Cleaved caspase 9
17
Cleaved caspase 3
37
GAPDH
D
Relative abundance (p53/GAPDH)
***
****
Relative abundance (Bax/GAPDH)
2.0
1.5
1.0
0.5
0.0
****
****
Relative abundance (Bcl-2/GAPDH)
**
***
Relative abundance (Cleaved caspase 9/GAPDH)
****
****
Relative abundance (Cleaved caspase 3/GAPDH)
****
****
Scramble siRNA
USP11 siRNA #1
USP11 siRNA #2
UA

Supplementary Figure S2. Knockdown of USP11 by two independent siRNAs transfection suppresses p53 expression and apoptosis in HK-2 cells.

To suppress USP11 expression, two independent siRNAs targeting USP11 (USP11 siRNA #1 and USP11 siRNA #2) were designed and individually transfected into HK-2 cells. (A) Cell lysates were subjected to immunoblot analysis with antibodies against USP11 and GAPDH. (B) Expression levels of USP11 were quantified by densitometry and normalized with GAPDH. (C) Cell lysates were subjected to immunoblot analysis with antibodies against p53, Bax, Bcl-2, Cleaved caspase 9, Cleaved caspase 3 and GAPDH. (D) Expression levels of p53, Bax, Bcl-2, Cleaved caspase 9, Cleaved caspase 3 were quantified by densitometry and normalized with GAPDH. Data were expressed as mean ± SEM (n=4 for each group). **P <0.01, ***P<0.001, ****P<0.0001.

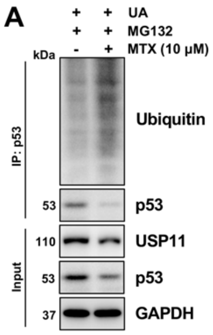
A
+ + UA
+ + MG132
- + MTX (10 μM)
kDa
IP: p53
Ubiquitin
53
p53
Input
110
USP11
53
p53
37
GAPDH

Supplementary Figure S3. MTX treatment enhances p53 ubiquitination and decreases p53 protein stability in HK-2 cells.

(A) Cell lysates were immunoprecipitated with anti-p53 antibody. The immunoprecipitates and input control were then subjected to immunoblot analysis with antibodies against Ubiquitin, USP11, p53 and GAPDH.

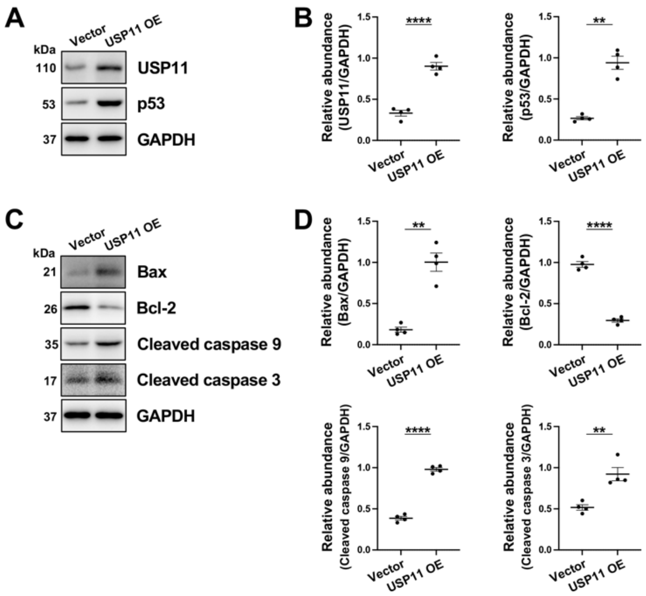
A
Vector
USP11 OE
kDa
110
53
37
USP11
p53
GAPDH
B
Relative abundance (USP11/GAPDH)
Relative abundance (p53/GAPDH)
****
**
Vector
USP11 OE
C
21
26
35
17
37
Bax
Bcl-2
Cleaved caspase 9
Cleaved caspase 3
GAPDH
D
Relative abundance (Bax/GAPDH)
Relative abundance (Bcl-2/GAPDH)
Relative abundance (Cleaved caspase 9/GAPDH)
Relative abundance (Cleaved caspase 3/GAPDH)

Supplementary Figure S4. Overexpression of USP11 upregulates p53 expression and promotes apoptosis in HK-2 cells.

HK-2 cells were transfected with USP11 pcDNA3.0 plasmid (USP11 OE). (A) Cell lysates were subjected to immunoblot analysis with antibodies against USP11, p53 and GAPDH. (B) Expression levels of USP11, p53 were quantified by densitometry and normalized with GAPDH. (C) Cell lysates were subjected to immunoblot analysis with antibodies against Bax, Bcl-2, Cleaved caspase 9, Cleaved caspase 3 and GAPDH. (D) Expression levels of Bax, Bcl-2, Cleaved caspase 9, Cleaved caspase 3 were quantified by densitometry and normalized with GAPDH. Data were expressed as mean ± SEM (*n*=4 for each group). **P <0.01, ****P<0.0001.
